# Development of an accreditation model for health education and promotion programs in the Iranian primary healthcare system: a Delphi study

**DOI:** 10.15171/hpp.2018.20

**Published:** 2018-04-18

**Authors:** Farid Gharibi, Jafar Sadegh Tabrizi

**Affiliations:** ^1^Iranian Center of Excellence in Health Management, School of Management and Medical Informatics, Tabriz University of Medical Sciences, Tabriz, Iran; ^2^Tabriz Health Services Management Research Center, Health Management and Safety Promotion Research Institute, Tabriz University of Medical Sciences, Tabriz, Iran

**Keywords:** Accreditation, Standards, Health Education and Promotion

## Abstract

**Background:** Considering the lack of accreditation models for health education and promotion(HEP) activities in the Iranian primary health care (PHC) system we conducted the present study to develop a national accreditation model for HEP actions in the Iranian PHC system.

**Methods:** After a comprehensive review on the accreditation models in PHC field, especially those concentrated on the HEP programs, an initial HEP accreditation model was developed.Then, applying the Delphi technique, 18 experts in the Iranian PHC system with field experience in HEP programs were invited to assess the initial model. In the two-round Delphi study,aggregation was provided on the opinions and the standards and indicators were finalized.Conventional content analysis was applied to make sense of the data collected in the study.

**Results:** The developed HEP accreditation model encompassed 62 indicators and five standards.The standards were as follow: "resources for HEP programs", "educational needs assessment of the target groups", "methods of providing a community with education", "management of health volunteers’ actions" and "evaluation of HEP programs".

**Conclusion:** The standards and indicators found in the present study may serve as an educational rationale for health educators while designing high-quality health education/promotion programs. This model may be helpful for health policy-makers and stakeholders while planning to assess the continuous quality improvement of HEP services delivered in the PHC systems.

## Introduction

Health education and promotion (HEP) is one of the most important and high-priority dimensions of public health which covers a wide range of activities with an increasing global notice.^1^ HEP is the science of making socio-behavioral changes which applies behavioral sciences to make optimal health changes.^2^ In fact, health educators are required to provide effective health educations and to improve self-care capacity in the community with the hope to bring about positive individual, organizational and social changes related to health.^2^ Capacity-building for HEP, to improve community health and reduce health inequalities, is a major challenge for many countries throughout the world. Many countries, especially European countries, have used accreditation to build capacity for HEP, because accreditation leads to capacity-building in human resources through facilitating positive changes in the system and enhancing cooperation.^1,3^

Accreditation is a public recognition on the level of achievement to the standards and essential functional requirements in an organization.^3^ Accreditations in HEP activities are developed and implemented to meet the existing health needs. In such accreditations, the aim is to improve quality of and to, strengthen and harmonize the activities related to HEP.^4^ For instance, despite the ongoing growth and diversity in HEP services throughout the Europe, no individual or institution was accountable to ensure the quality of provided educations and professional activities.^1^ Therefore, the International Union for Health Promotion and Education (IUHPE) launched an accreditation system for educational programs and activities in Europe.^4^

Nowadays, development and implementation of accreditation programs for quality promotion and professional development are indispensable necessities for capacity-building of human resources in HEP.^1^ The development of such accreditation systems may provide a unique opportunity for capacity-building, education and research in HEP field, and may also ensure the quality of delivered services.^1,5^

Iran as a country located in the Eastern Mediterranean Region (EMR), has a wide primary health care (PHC) system.^6,7^ In the recent decades, PHC in Iran has been a successful system in raising the level of health and life expectancy in the community. This system, however, has had major challenges including the decline in the quality of health services and the reduction in the acceptability and use of services.^8-13^ Such challenges may be due to the factors like population growth, changing in the population pyramid, lifestyle changes, changes in disease patterns and the inability of the PHC system to effectively and efficiently respond to the changes.^8-13^

As accreditation may be one of the most important tools in improving the quality of health services and promoting the performance of health centers, it seems that developing and implementing HEP accreditation programs in the Iranian PHC system will result in quality improvement in the health promotion services. Utilizing accreditation programs in HEP field may has major benefits including the commitment of healthcare centers to improving the quality of their services, ensuring the quality of provided programs by conducting external evaluation, ensuring the professional competencies of HEP personnel, increasing the ability of healthcare centers to apply for a maximum level of funds, making links between the HEP field and other healthcare fields, accelerating and facilitating positive changes in health programs and systems, and increasing public trust in the services provided by the HEP section.^5^

Considering the lack of such accreditation models in the Iranian PHC system, especially in HEP field, we conducted the present study to develop a national accreditation model for HEP actions in the Iranian PHC healthcare system.

## Materials and Methods

Accreditation may result in improvement in performance and quality of services only if appropriate and evidence-based assessment standards are developed and applied.^14^ Accreditation standards should also reflect its objectives and cover the key processes and activities of a health system.^14^ Hence, the first step in development of efficient and effective accreditation standards is to determine valid sources and methods.^15,16^

In order to develop accreditation standards in the HEP field different methods have been applied including literature review on the existing accreditation models, comprehensive studies on the nature and different aspects of HEP and investigating the ideas of experts in designation of HEP indicators/measures.^17^ Accordingly, the research team in our study has developed primary standards based on the existing HEP literature and the documents in the HEP field of PHC services, the ideas of HEP expert panel members and comprehensive review of successful accreditation models worldwide and in the EMR.^18-20^ We also reviewed educational models in the field of higher education, in particular medical education, such as the Tennessee Educational Assessment Model.^21^

After development of the primary standards from the abovementioned sources, Delphi technique was used to reach consensus on the developed standards/measures. Two indicators of importance and feasibility on a 9-degree scale was applied in the Delphi method.^15^

In order to implement this technique, all the primary standards/measures were included in the Delphi questionnaire and were assessed by a team of experts. In order to implement a Delphi method, there is a need for at least 10 experts to participate in the study.^22,23^ In the present study, 18 experts with more than 5 years of field experience in HEP programs were invited to participate in the study. The questionnaires were delivered to the experts in two ways: provide the available experts in person with a hard copy of the questionnaires and submitting a soft copy of the questionnaires to the remote experts in the other provinces via email. After analyzing the results at each stage, the questionnaires were prepared for the next stage and presented to the experts. This process was continued until the final agreement on the standards was obtained. In the two rounds of the study, 16 experts completed and sent back the questionnaires. So, the response rates for the first and the second rounds were 88.88% and 100%, respectively.

In the analysis phase of the study, the median index was considered as the basis for decision on an item. This index is not affected by unconventional and extreme cases. So, if the median for an item was between 1 and 4, the standard was excluded from the study. If the median index was at a range between 4 and 7, then the standard was considered to be entered at the next stage. Also, if the index was higher than 7, so the standard was directly accepted and entered in the final model.^24^ As a noteworthy strategy we used in this section, we provide the experts with feedback on the results obtained from the previous rounds (the total median) and the scores given to the standard by each expert. This strategy entailed more reflections by the experts on the scoring, modification and adjusting of the score given to an item. In the consensus process, if changes in the scores given by the experts in two consecutive rounds of the study for each item were less than 15% of the total median score, then the standard was regarded as an agreed item and therefore was not entered at the next round of the study.^25^

## Results

Our aim in this Delphi study was to develop a national accreditation model for HEP actions in the Iranian PHC healthcare system. We found the main focus of the expert panels to be provision of resources for health educators (in particular qualified health educators), the processes of conducting educational programs, and conducting evaluation programs for educational plans aiming to assess the effectiveness of the plans in solving community health problems.

Our review on the comprehensive accreditation models in the field of PHC showed neglect in accrediting HEP programs and, therefore, a lack in accreditation standards for this field. However, a few accreditation models were found in which this field of PHC system was partially considered as a part of the model. Interestingly a majority of these models were from the EMR countries including Egypt,^18^ Jordan^19^ and Saudi Arabia.^20^ The criteria that these accreditation models were superficially addressed in their standards included learning processes, the resources used for health education and the evaluation of health education programs.^18-20^

After a review with more details on the accreditation models in the HEP field, we found that their main focus was on empowering human resources^4,5^ with the hope to provide effective education programs for the target community.^18-20^ As an example, the European accreditation model for HEP was designed and implemented after a full consideration on the necessary competencies of health educators, professional health promotion standards and accreditation frameworks.^4^ The focuses of this model was on various areas such as improving the quality of activities in HEP field, human resource empowerment, as well as the interests and motivation of human resources in this area.^4^ In the HEP accreditation model of the United States also there are emphasizes on the responsibilities of health educators in critical aspects like conducting educational needs assessments, designation and implementation of effective educational strategies and interventions programs and scientific research in the field of HEP.^26^

Moreover, several scientific management indicators of HEP programs were found as criteria for accreditation in HEP field. These criteria included development of mission, perspective, goals and strategy for HEP programs; scientific and effective evaluation of health centers and their programs; development of educational contents and curriculum based trainings to promote the level of self-care behaviors among different populations.^5^ In addition, the needs assessment-based development of health policies, self-assessment and self-evaluation (particularly on the effectiveness of the undertaken activities), development of appropriate and need-based educational contents and messages, adequacy of health promotion instructors, effective participation of health personnel in the activities related to HEP were emphasized in the literature.^27^

As the nature of the HEP services is focused on education, we selected the Tennessee Medical Education Model as the basic framework.^21^ So the domains of this model as well as those found in the other PHC accreditation models were applied to develop the HEP accreditation standards for Iranian PHC system. Accordingly, the main dimensions of the model such as needs assessment of a target community, development of needs assessment-based educational contents, learning /teaching processes, educational evaluation and quality assurance were studied and exploited.^21^

Based on the abovementioned findings, five main dimensions were considered as accreditation standards for HEP field: “resources for HEP programs”, ”educational needs assessment of the target groups”, ”methods of providing a community with education”, ”management of health volunteers’ actions” and ”evaluation of HEP programs”. These standards comprised 62 indicators.

At the first round of the Delphi study, a questionnaire consisting 62 indicators in 5 dimensions was presented to 18 PHC experts in the field of HEP affiliated to health departments in different Iranian medical universities. Sixteen experts completed and sent back the questionnaires (response rate = 88.88%). After initial analysis, 51 indicators were approved as they were found with, at least, the minimum score of 7 in both dimensions of importance and feasibility. Eleven indicators were considered to be sent to the second round due to the score of 4 to 7 in one or both criteria. Thus no item was found to be excluded from the study.

In the second round, the remaining 11 indicators were again sent to the experts. Along with these indicators, the responses of all experts on these indicators, the total scores and the score of each expert on the indicators were individually reported to the experts. At this stage, the experts reviewed and re-scored the indicators and sent back their responses to the researchers. Analyzing the responses, we found all the standards with the minimum score required for approval. During the rounds of the study, all the revisions suggested by the panel, which were mainly on wording and phrasing the items, were applied to the items. The selected indicators and their scores on importance and feasibility as well as the final median scores for all the items are shown in Table 1.

In terms of importance, the dimensions of “resources for HEP programs” and “management of health volunteers’ actions” were found with the highest (8.32) and the lowest (7.45) average scores, respectively. In terms of feasibility, the dimensions of ”methods of providing a community with education” and “educational needs assessment of the target groups” were found with the highest (8.14) and the lowest (7.21) scores, respectively. The total average scores for all standards in terms of importance and feasibility were found to be 8.06 and 7.53, respectively.

## Discussion

Our aim in this study was to develop a national accreditation model for the programs implemented in the HEP field of the Iranian PHC system. We found the final HEP accreditation model with five main dimensions including “resources for HEP programs”, “educational needs assessment of the target groups”, “methods of providing a community with education”, “management of health volunteers’ actions” and “evaluation of HEP programs”. In Iran, considering the large population, the geographical extent and the diversity, it seems to be necessary to address all relevant dimensions of the HEP activities.

The first standard was “resources for HEP programs. This dimension included the resources needed to provide HEP programs for a community which emphasizes the availability of human resources, monetary issues, information infrastructures, educational spaces, and equipment and materials with acceptable quality and quantity. These issues were emphasized in the European accreditation model of HEP.^4^ The documentation for this standard was obtained from the HEP programs implemented in the Iranian PHC system and through the interviews with the Iranian PHC system experts.

The focus in the second standard, namely “educational needs assessment of the target groups”, is on the educational needs of a target community which refers to the components like logical grouping of a target community, identifying the educational needs assessment of each group, prioritizing the needs, and developing high quality educational contents tailored to the identified needs. All these criteria were highlighted in the HEP Model of the United States^26^ and Taiwan^27^ as well as the Tennessee Medical Education Model^21^ and the interviews with scholars in the present study.

The third standard was “methods of providing a community with education”. This dimension comprised a series of indicators like the necessity of teaching systematic and scientific materials to a community, the application of collaborative and active learning methods, step-by-step and targeted learning, considerations on the characteristics of a target group before and while learning, exact timing and suitable methods for training a target group, joining influential people in the teaching process for a community, respecting cultural values ​​of a community in health educational promotion programs and scientific and precise evaluation of the educational processes and its effects. These indicators were also highlighted in the integrated accreditation models designed the Eastern Mediterranean countries including Egypt,^18^ Jordan^19^ and Saudi Arabia^20^ as well as the Tennessee Medical Education Model^21^ and the documentations from the PHC system of Iran.

The dimension of “management of health volunteers’ actions” has focus on health volunteers, who are elected among the volunteer individuals in a community to provide the people in a community with applied and face-to-face HEP actions. In this dimension of the model there are emphases on identifying the educational needs of health volunteers, academic empowerment and skills improvement, motivating the volunteers for continuing their activities and evaluating and continuously improving their performance, as well. This standard and its related indicators are specific to Iranian PHC system and were derived from the documentations of Iranian PHC system and the interviews conducted with the experts.

The fifth standard called “evaluation of HEP programs” was also related to the effectiveness of the HEP programs and the level of participation from the individuals in a community in the health activities. This standard also included the impacts of HEP programs on improving knowledge, attitude, skills and self-care behaviors in a population and their level of satisfaction with the HEP services. This standard and its related indicators were derived from the accreditation model of USHEP^26^ and Taiwan,^27^ as well as the Tennessee Medical Education Model,^21^ and the interviews conducted with the experts.

Assessing the HEP accreditation programs indicated that the EMR accreditation models were more comprehensive than other programs. In the European model,^4^ there was a highlight on the “competencies of health providers”, and in, the US model^26^ an emphasis was placed on “educational needs assessment of the target groups” and “evaluation of HEP programs”. However, in the Egypt,^18^ Jordan^19^ and Saudi Arabia^20^ models, enough attention was comprehensively paid to all aspects of HEP activities. As our model in the present study was developed based on these reference models, we can claim that our model, in terms of structure and comprehensiveness, has a level of similarity with the EMR models, especially with those of Egypt^18^ and Saudi Arabia.^20^

The Egypt PHC accreditation model^18^ covers the domains such as “identifying and covering community health education needs”, “proper training of the HEP personnel”, “providing proper educational location, materials and teaching tools”. The standards in the Jordanian PHC accreditation model^19^ are emphasized on “HEP service delivery to targeted population especially high-risk groups”, “developing and using educational materials in an understandable format for target population” and “participation of HEP personnel in a variety of evidence based health promotion and disease prevention programs”. Also, the HEP accreditation model of Saudi Arabia^20^ are accentuated on “community participation”, “health promotion and education plan”, “relevant health promotion and education programs”, “staff competency” and “performance measurement and improvement”.

Our results in the Delphi study showed the dimensions of “resources for HEP programs” and “management of health volunteers’ actions” as the standards with the highest and the lowest levels of importance, respectively. The high importance that the experts gave to the resources in HEP may be due to the tangibility they have perceived in the resource-related measures and the shortcomings exists in this area. Also, the low score of importance they considered for health volunteers may be attributed to the low effectiveness of the trainings provided by them compared to other health educators and/or to the fact that the health volunteers program has been less canonized in the recent years. The experts also considered the dimensions of “methods of providing a community with education” and “educational needs assessment of the target groups” as the standards with the highest and the lowest feasibility. This finding may be attributed to the common role of community education methods and the novelty of educational needs assessments especially in a scientific manner.

As the final step in developing an accreditation model is to conduct a pilot test to identify the weaknesses and strengthens in practice, we suggest the implementation of model applying scientific principles and valid tools. Obviously, active participation of PHC experts, health policy makers and stakeholders at this step will guarantee the applicability and acceptability of the model. We also suggest the application of this model in HEP initiatives with the hope to improve the quality and effectiveness of HEP services provided to the community. In addition, due to the lack of similar accreditation models in other fields of the Iranian PHC system, the need for similar studies to develop accreditation models for all specialized areas seems to be necessary.

One of the strengths in our study was the development of a unique and comprehensive accreditation model for HEP programs in the PHC system in Iran. Validating the initial model through the Delphi study and incorporating the ideas of PHC system experts in this validation process was other strengths for this study. Moreover, the response rate of the experts in the Delphi study was high which assured the development of a strong and acceptable model from the experts’ point of view. Also, confirmation of all standards and indicators by the experts in terms of importance and feasibility, ensured the reliability of the model.

A main limitation of the present study was the use of Delphi method. Although the Delphi technique is a valid and reliable method to obtain consensus, but has some complications as follow: difficulty in selecting the participants (finding both famous and knowledgeable experts), ensuring the anonymity of the participants and the management of extreme responses.^23^

## Conclusion

Our findings in this accurately conducted Delphi study provided a transparent HEP accreditation model with five standards and 62 indicators for the Iranian PHC system. This model may help health policy makers and stakeholders while planning to assess the quality of HEP services delivered in the PHC systems. Such models in the field of health promotion may ensure continuous quality improvement of HEP programs. It may be concluded that the standards and the indicators found in the present study may serve as an educational rationale for health educators and health promoters while designing high quality health education/promotion programs.

## Ethical approval

This study was approved by Ethics Committee in Tabriz University of Medical Sciences (Ethics No. 28/3/1392).

## Competing interests

The authors declare that they have no competing interests.

## Authors’ contributions

FG and JST contributed equally to the work of the manuscript because they jointly designed and implemented the study, wrote, edited and finalized the manuscript.

## Acknowledgements

The research team is very grateful to the sincere cooperation of the experts who participated in the specialized meetings or responded to the Delphi survey despite their busy schedule. The team is also thankful to Tabriz Health Services Management Research Center for funding the research.

**Table 1:** Selected indicators and their scores on importance and feasibility

**No.**	**Measures**	**1st round**	**2nd round**	**Importance**	**Feasibility**
**Standard 1:** resources for HEP programs					
1-1	The personnel required to carry out activities related to HEP have been hired with a reasonable quantity.	✔		7.5	7
1-2	The personnel of this field have received proper start and in-service trainings.	✔		8	9
1-3	The personnel required to carry out activities related to HEP have sufficient skills and experience to perform the assigned tasks.	✔		8.5	7.5
1-4	The funds required for HEP are provided and used in an appropriate manner.	✔		9	8.5
1-5	Health centers spend their funds according to a justifiable approach based on health priorities.		✔	7	8.5
1-6	Health centers have made viable investments in creating and expanding information management infrastructure such as computer systems, internet access, software and programs.	✔		8.5	8
1-7	The health centers, in collaboration with higher management bodies, have made viable investments in the provision of portals, websites and their databases.		✔	7.5	8
1-8	The centers of comprehensive health services and higher levels of management are capable of generating, storing and disseminating reliable information through creating the needed infrastructure.	✔		7	7.5
1-9	Health centers, in collaboration with higher management bodies, have a good performance in the production and dissemination of health information.	✔		8.5	9
1-10	Physical spaces in health centers are provided and used appropriately for HEP activities.		✔	8.5	8.5
1-11	The required educational equipment is used properly in HEP activities.	✔		9	8
1-12	The raw materials used in the delivery of the service, especially the stationeries and reproduction facilities, are provided and used in an appropriate manner.	✔		9	8.5
**Standard 2:** educational needs assessment of the target groups					
2-1	The covered society is clustered based on age, sex, demographic information, occupational status and health level, and accurate statistics are provided for each of them.	✔		7	8
2-2	Educational needs of all target groups are determined precisely on the basis of health indicators, available scientific evidence, interviews with experts, and opinions of the community.	✔		7.5	8
2-3	In carrying out this needs assessment, the epidemiological and demographic indicators, community expectations, higher level documentation and the nature of the target groups are considered.	✔		8	7.5
2-4	The specified needs are prioritized and estimated on the basis of indicators such as importance, urgency, and operational capability.	✔		8	7.5
2-5	Educational needs assessments are conducted periodically and by experts.	✔		8.5	7
2-6	The educational content is based on the needs assessments and reliance on scientific data and existing applied experiences.	✔		7.5	8.5
2-7	Efficient databases are made from educational content related to this field and updated in appropriate intervals.		✔	8	9
2-8	The content of educational materials is tailored to the interests, health knowledge and mental, psychological and social development of the audience.	✔		8	7.5
2-9	The quality of provided educational content is periodically evaluated by specialized teams.	✔		7	7
2-10	The practical empowerment of the community to promote self-care is the goal of developing all educational content.	✔		9	9
2-11	Health education experts’ access to information infrastructure and educational content is facilitated.	✔		7.5	7.5
2-12	Public access to scientific resources and educational content has been facilitated in an effective way through various channels.		✔	7	8
2-13	Public education calendar is based on health events and scientific needs assessments.	✔		7.5	7.5
2-14	The educational programs specified in the health calendar are informed and implemented in the appropriate manner to target groups.	✔		8	8
**Standard 3:** methods of providing a community with education					
3-1	The health education process is done step by step and continuously to improve the health behaviors of the community.	✔		8.5	7.5
3-2	Active and effective teaching methods that are appropriate to the characteristics of target groups are used in the design and implementation of educational programs.	✔		8	7.5
3-3	The scheduling of educational programs is defined and communicated in a rational way and in accordance with the conditions of target groups.	✔		8	7
3-4	Positive and mutual interaction between educators and learners is considered seriously.	✔		7.5	7
3-5	>Health education programs support all other areas of PHC.	✔		7.5	7
3-6	The interest group of the community is used in order to have a more effective education.	✔		9	8
3-7	The available media are used to provide public education.		✔	7	7
3-8	Community members can easily ask their health care providers about their questions and concerns.	✔		8	8.5
3-9	The community members receive appropriate and accurate answers to their questions and concerns from health providers.	✔		8.5	8.5
3-10	The health education instructor refuses to confront values, beliefs, customs, and common traditions.		✔	7.5	7
3-11	The health education instructor respects the right of choice and discretion of his or her audience with dignity and gives them the opportunity to analyze, adapt, select and choose the health behaviors.	✔		8	7
3-12	Self-care principles in all areas of health are taught to the various classes of society in a simple language.	✔		8.5	7.5
3-13	Appropriate developmental and final evaluations are conducted to examine the effectiveness of educational programs.	✔		9	9
3-14	The quality of the educational process is evaluated and audited periodically by specialized teams.	✔		8.5	9
3-15	Educational programs are evaluated by receiving knowledge, attitude and skill feedback from the learners.	✔		8.5	8
3-16	The results of evaluations are used to improve educational programs.	✔		7	7.5
**Standard 4:** management of health volunteers’ actions					
4-1	Health volunteers are selected from among the most capable and motivated people who have a good interaction with the community.	✔		8	8
4-2	The ratio of the number of health volunteers to rural households (one for every 10 to 30 rural households) is determined in an appropriate manner.		✔	7	7
4-3	Constant and appropriate assessments are made on the educational needs of health volunteers.	✔		7.5	8
4-4	Health volunteers take early and in-service training courses based on the nature of their duties and the designated information needs.	✔		7.5	7.5
4-5	Educational content for training the volunteers is determined carefully, based on their and the community’s information needs.	✔		8.5	9
4-6	Health volunteers are trained according to the specified educational content with high accuracy and quality.	✔		9	8.5
4-7	Appropriate indicators are determined for evaluating the volunteers' activities.	✔		9	8.5
4-8	Activities of health volunteers are analyzed based on the information obtained.	✔		9	8
4-9	Proper and precise interventions are being developed and implemented to improve the performance of volunteers based on evaluations carried out.	✔		8	7
4-10	Effective financial and spiritual motivations for the quantitative and qualitative improvement of health volunteers' activities are determined and implemented.		✔	7	7.5
4-11	All the required facilities are available to volunteers in order to provide appropriate educations to them.	✔		7.5	7
4-12	Assessments have shown that there are continuous improvements in the indicators related to the activity of health volunteers.	✔		7	7.5
**Standard 5:** evaluation of HEP programs					
5-1	The public belief in the society is that people's lifestyle can affect their health.	✔		8.5	8.5
5-2	The people of the community are equipped with the necessary knowledge and skills in a way that can contribute to their health issues.	✔		8	8
5-3	People actively participate in the community's health activities.		✔	7.5	7
5-4	All people in the community, especially vulnerable and at-risk groups, have the knowledge, attitude and skills and appropriate self-care for the prevention of various diseases.	✔		7.5	8.5
5-5	Patients, especially those with chronic illness, have knowledge, attitude and self-care to control their disease.	✔		9	8.5
5-6	The trainees are increasingly satisfied with different aspects of the courses provided.		✔	7	7.5
5-7	The covered communities, especially those at risk, use appropriate health behaviors to avoid illness or control it effectively.	✔		8.5	9
5-8	The positive health indicators indicate the desirable effectiveness of health education activities.	✔		8	7
